# Reiki and Therapeutic Touch for symptom burden and quality of life in palliative settings: A systematic review

**DOI:** 10.1177/02692163261437606

**Published:** 2026-05-10

**Authors:** Raquel Pontes-Gomes, Paulo Reis-Pina

**Affiliations:** 1 Center for Palliative Medicine, Faculty of Medicine, University of Lisbon, Lisboa, Portugal; 2 Acute Palliative Care Inpatient Unit, Pulido Valente Hospital, Santa Maria Local Health Unit, Lisboa, Portugal

**Keywords:** complementary therapies, palliative care, quality of life, Reiki, symptom burden, therapeutic touch

## Abstract

**Background:**

Reiki and Therapeutic Touch are complementary therapies sometimes used in palliative and end-of-life care.

**Aim:**

To examine the available evidence regarding the effects of Reiki and Therapeutic Touch, compared to usual care, in palliative and end-of-life care.

**Design:**

Systematic review registered in PROSPERO (CRD420251059364; May 23, 2025).

**Data sources:**

MEDLINE, Web of Science, and Scopus were searched for English-language studies published between 2013 and 2024. Eligible studies included patients receiving palliative care who underwent Reiki and/or Therapeutic Touch compared with usual care. Any primary study design was eligible. Risk of bias was assessed and findings synthesized narratively.

**Results:**

Nine studies involving 415 participants were included: five mixed-methods studies, three randomized controlled trials, and one qualitative cross-sectional study conducted in North America (*n* = 6) and Europe (*n* = 3). Cancer was the predominant diagnosis. Risk of bias raised some concerns in randomized trials and was moderate to high in qualitative and non-randomized designs. Some studies reported improvements in symptoms (pain, anxiety, depression, fatigue, and stress), and in quality-of-life domains (sleep, relaxation, energy, hope, and emotional well-being). Qualitative findings described perceived relaxation, comfort, and emotional support. Reporting of harms was limited; only one study monitored adverse events and reported one serious event considered potentially related to the intervention.

**Conclusions:**

Evidence regarding Reiki and Therapeutic Touch in palliative and end-of-life care remains limited and heterogeneous. The very low certainty of evidence precludes firm conclusions regarding their effectiveness. Further well-designed studies are needed to clarify their potential role in palliative care.

**What is already known on the topic?**

- Reiki and Therapeutic Touch are complementary therapies increasingly used in palliative and end-of-life care.
- Existing evidence remains limited, remains methodologically heterogeneous, and findings are often inconclusive.

**What this paper adds?**

- This systematic review included nine original studies involving 415 participants, most of whom had cancer.
- Some studies reported improvements in symptoms such as pain, anxiety, depression, fatigue, and stress.
- Improvements in quality of life—including sleep quality, relaxation, energy, hope, and self-confidence—were also described.

**Implications for practice, theory, or policy**

- Reiki and Therapeutic Touch may be considered complementary interventions within holistic palliative care approaches.
- These therapies may contribute to person-centered care by addressing physical, emotional, and spiritual needs.
- Further well-designed research is required to clarify their potential role in palliative care.

## Introduction

### Rationale

In both developing countries and some industrialized nations, a significant portion of the population relies on complementary and alternative medicine.^1^ This reliance may reflect growing acceptance of holistic approaches in health care, but may also relate to perceived gaps in conventional care or limited access to supportive resources.

Palliative care is a holistic approach aimed at alleviating health-related suffering and improving the quality of life of patients and their families.^2^

Reiki and Therapeutic Touch may be incorporated into integrative or hospice care programs and are intended to complement, rather than replace, conventional medical treatment.^3^

Reiki and Therapeutic Touch were considered together in this review because both are typically classified as biofield-based complementary therapies^4^ and are implemented in similar clinical contexts. However, the clinical mechanisms underlying these therapies remain debated, and reported benefits may reflect non-specific factors such as therapeutic interaction, relaxation responses, or placebo effects.^3,5^

Previous reviews have suggested that some complementary therapies may be associated with improvements in physical or psychosocial symptoms in hospice populations, although the available evidence is limited and randomized controlled trials remain scarce.^6^ Another review including quasi-experimental studies suggested that Reiki and therapeutic massage may help alleviate symptom clusters in pediatric palliative care, but the overall risk of bias was moderate.^7^

Given the increasing emphasis on patient-centered care, there is growing interest in supportive interventions that may improve symptom control and quality of life in palliative settings.^8^ In this context, and considering the ongoing debate regarding the mechanisms and effectiveness of biofield-based therapies, it is important to critically examine the available evidence regarding Reiki and Therapeutic Touch and their potential effects on symptom burden and quality of life in palliative care settings.

### Objectives

This systematic review aimed to examine the available evidence regarding the effects of Reiki and Therapeutic Touch, compared with usual or standard care, on patient outcomes in palliative and end-of-life care.

## Methods

This systematic review followed the recommendations of the Cochrane Handbook for Systematic Reviews of Interventions,^9^ and was reported in accordance with the Preferred Reporting Items for Systematic Reviews and Meta-Analyses guidelines.^10^ The initial version of the protocol was registered in PROSPERO, after the initiation of the search but before data extraction began.

### Eligibility criteria

*Population*- Patients of any age or gender with advanced, incurable diseases enrolled in palliative care and/or end-of-life care programs.

*Intervention*: Reiki and/or Therapeutic Touch.

*Comparator*: Usual or standard care, or no control group where comparators were not reported.

*Outcomes*: At least one of the following: symptom management/burden or quality of life. No predefined definition of quality of life was imposed; studies were eligible if they assessed quality of life as defined by their own authors using patient-reported or validated quality-of-life measures.

*Study design*: Any study design was eligible.

*Exclusion Criteria*: Records were excluded at the initial screening stage if they were written in languages other than English; if they were literature reviews, study protocols, protocol descriptions, conceptual models, conference abstracts, posters, book chapters, letters, editorials, commentaries, academic theses, or proposals and calls for collaboration. These types of publications were excluded *a priori* because some of them are not consistently peer-reviewed and typically provide insufficient methodological or outcome information for robust evaluation. Our review therefore focused on full-text, peer-reviewed articles to ensure adequate methodological detail for appraisal and synthesis.

### Information sources

The literature search was conducted in the MEDLINE (via PubMed), Web of Science, and Scopus databases for English-language articles published between January 1, 2013, and December 31, 2024. The last database search was performed on January 3, 2025.

The reference lists of included studies and relevant systematic reviews or meta-analyses were manually screened to identify any additional potentially eligible studies.

### Search strategy

The search combined free-text terms and database-specific headings. The following search strategy was applied: (“palliative care” OR “terminal care” OR “hospice care” OR “end of life care”) AND (“therap* touch” OR “reiki”). Filters: English language; publication years 2013–2024. The full search strategy for each database is presented in Supplemental Table 1. Only publications in English were considered, as this is the predominant language of publication in international palliative care and complementary therapy research. Automated translation tools (e.g. Google Translate, ChatGPT) were not used, as systematic reviews require precise interpretation of methodological and statistical details that may not be reliably captured through machine translation. The search was limited to studies published between January 1, 2013, and December 31, 2024. This decision was made a priori to update a prior narrative review^11^ that had synthesized 30 studies published between January 2008 and June 2013, and to capture more recent evidence reflecting contemporary clinical and research contexts. In addition, the time restriction was applied to maintain feasibility, as the review was conducted by two researchers without external funding.

### Selection process

First, titles and abstracts of publications were screened for relevance. Potentially eligible articles were then selected for full-text analysis. The final selection was based on study design and relevance to the review. Both authors independently conducted the selection process, and no automation tools were used.

### Data collection process

A data-charting form was created using Microsoft Excel (Microsoft Corp, 2016) and iteratively refined through testing. Data were collected for each report by two independent reviewers. Any discrepancies between reviewers were resolved through discussion until consensus was achieved. Original authors were not contacted for additional data or confirmation. No automation tools were employed in this process.

### Data items

Data were extracted on symptom management/burden and quality of life. Additionally, information was collected on: (a) Study characteristics (e.g. authors, country of origin, year of publication); (b) Study design; (c) Objectives; (d) Population characteristics; (e) Intervention details; (f) Control/comparator group details; (g) Outcome measures; (h) Scales and/or assessment tools used; (i) Main findings.

During revision of the manuscript, the included studies were also re-examined for any reporting of adverse events, harms, or safety outcomes associated with the interventions.

### Study risk of bias assessment

Two independent reviewers assessed the quality of the included studies, resolving any discrepancies through discussion until consensus was achieved. For each domain, judgments were based on the algorithms recommended by the RoB 2 Cochrane tool for randomized controlled trials,^12^ the Joanna Briggs Institute Critical Appraisal Checklist for qualitative research,^13^ and the Mixed Methods Appraisal Tool for studies with both qualitative and quantitative components.^14^

For qualitative studies, overall quality was summarized using the proportion of “Yes” responses relative to the total number of items in the checklist. Because the Joanna Briggs Institute tool does not recommend standardized cut-offs for overall quality classification, thresholds (low < 0.5; moderate 0.5–0.7; high > 0.7) were defined by the authors to facilitate consistent interpretation across studies.

### Effect measures

Effect measures were reported as defined in the included studies. Because of heterogeneity in study designs, outcome measures, and reporting methods, effect measures were extracted and presented as reported by the original authors without statistical transformation. These measures were based on the outcome instruments used in the primary studies, which generally included patient-reported or clinically recognized measures of symptom burden and quality of life.

### Synthesis methods

A meta-analysis was not conducted in this review. Meta-regression was also not feasible due to: (a) the limited number of included studies and (b) substantial heterogeneity across study populations, interventions, and outcomes, which precluded the generation of a meaningful and valid summary. Likewise, no formal evidence grading framework was applied; instead, study findings were synthesized narratively.

For the synthesis, extracted data were categorized into quantitative outcomes (e.g. symptom burden and quality of life), qualitative outcomes describing participant experiences, and any reported adverse events, harms, or safety outcomes associated with the interventions.

To facilitate interpretation, the findings are summarized in tables describing study characteristics (Table 1), reported outcomes (Table 2), risk-of-bias assessments (Tables 3 and 4), and certainty of evidence assessments (Table 5).

**Table 1.** Characteristics of the included studies (*n* = 9).

Authors; country; year	Study design	Objectives	Sample	Setting	Interventions	Control	Main outcomes	Scales used	Comments
Kirshbaum et al.; United Kingdom; 2016.^18^	Cross-sectional qualitative study (a framework analysis)	-To explore the perceptions and experiences of reiki for cancer women. -To identify outcome measures for an intervention study.	*n* = 10 women, mean age 63 years	The Kirkwood Hospice (Huddersfield); the Overgate Hospice (Halifax) and the Alexandra Huddersfield Spa.	-Semi-structured interviews that were audiotaped, transcribed and coded using a framework analysis.	No control group.	Pain, sleep, symptom management, energy, wellness, calm and peace, depression, hope, self-confidence.	Exploratory research without objective scales.	Cancers: 30% lung, 30% breast, 10% endometrial, 10% sarcoma, 10% leukemia, 10% bowel.
Thrane et al. ; United States of America; 2021.^19^	Quasi-experimental pre-post mixed-methods 1-group pilot study	To assess feasibility and acceptability of using Reiki therapy for children ages 7–16 years old receiving PC.	*n* = 32 −16 children (11 females, 5 males, mean age 12.6 years), −15 mothers (mean age 43.7 years) -1 nurse (female)	Children recruited from Pediatric PC Service of Children’s Hospital, University of Pittsburgh Medical Center, Pennsylvania.	-Two 24-min Reiki therapy sessions done by a trained Reiki Master. -1–3 days between sessions. -After the second session: a structured interview was conducted with parents and children to assess acceptability and overall response.	No control group.	Relaxation, pain, calm, happiness, body temperature	Exploratory research without measurement tools or scales. Results come from patients and mothers’ descriptions of Reiki experience.	Children diagnosis: 44% cancer, 25% congenital condition, 31% genetic condition
Thrane et al.; United States of America; 2021.^20^	Single-arm, mixed-methods, quasi-experimental pre- and poststudy	To determine feasibility and acceptability of Reiki intervention with hospitalized children with chronic, life-limiting conditions receiving PC.	*n* = 32 −16 children (9 males, 7 females, mean age 2.2 years), −16 mothers (mean age = 29.5 years)	Children recruited from Pediatric PC Service of Children’s Hospital, University of Pittsburgh Medical Center, Pennsylvania.	Six 17-min protocolized Reiki sessions delivered as 2 sessions per week for 3 weeks.	No control group.	HR, RR, oxygen saturation, pain, and stress, child’s health status and QOL, parent’s health status and symptom profile (at baseline, end, and 3 weeks later).	-Feasibility was calculated by the percentage of families enrolled in the study. -Acceptability was assessed by the percentage of families who completed all measures and 5 out of 6 Reiki sessions.	Children diagnosis: cancer, meningitis, nontraumatic brain injury, respiratory failure or arrest, extreme prematurity, and congenital conditions.
Thrane et al.; United States of America; 2022.^21^	Mixed-methods study	-To evaluate the effects of Reiki in hospitalized young children. -To evaluate parent/guardian and staff perception of the child’s reaction to Reiki therapy. -To explore the stress/relaxation response of children during Reiki therapy.	*n* = 32 −16 children (15 females, 1 male, mean age 2.2 years), −16 mothers with mean age 29.5 years.	Children recruited from Pediatric PC Service of Children’s Hospital (365 beds), University of Pittsburgh Medical Center, Pennsylvania.	-Participants received two Reiki sessions per week for 3 weeks. -Physiologic measures assessed pre/post each session, and parent reports of pain and QOL were collected (at baseline, 3 weeks, and 6 weeks after). -The parent rating of Reiki’s perceived efficacy and their own symptoms.	No control group.	Pain, Stress; QOL, oxygenation, PROMIS-29, PTES.	-Pain (VAS and FLACC); -Stress (HR variability and RR); -QOL (PedsQL); -PROMIS-29; -PTES.	N/A
Utli et al. ; Turkey; 2023.^22^	Single-blinded randomized controlled study	To determine the effect of Accupressure or Reiki interventions on the levels of pain and fatigue of cancer patients admitted to PC clinics.	*n* = 156 Stage III/IV cancer -Acupressure group: *n* = 52 (36 males, 16 females), 51.9% aged 18–64 years. -Reiki group: *n* = 52 (29 females, 23 males), 55.8% aged 18–64 years.	PC clinics of two state hospitals of the Ministry of Health in a province in eastern Turkey.	-Acupressure or Reiki was applied to their intervention groups for a total of eight sessions of 20 min each over 4 weeks, once a day on 2 days a week. -After the procedure, the patient rested for 10 min.	-Control group: *n* = 52 (35 males, 17 females) 51.9% aged 18–64 years, 48.1% ⩾65 years	Pain, fatigue, and analgesic use.	AFF, NPRS BFI, Pain (every 4h using a VAS), weekly performance status (ECOG-PS).	-The control group was merely allowed to rest for 20 min. -There was someone present during their rest period.
Ünal Aslan and Çetinkaya ; Turkey; 2022.^23^	Randomized controlled experimental research	To investigate the effect of TT on spiritual care and sleep quality in patients receiving PC.	*n* = 73 (39 males, 34 females), mean age 80,5 years	PC service, Research Hospital, Turkey	-n = 36 (42,7% female, aged 75 years). −47.2% needed spiritual care. −41.7% had poor sleep quality. -A 15 min (3x/week for 1 month) session of TT on patients hands while trying to perceive individual’s energy flow and the patient’s responses.	-n = 37 (54,1% males, aged 76–80 years). −59.5% needed spiritual care. −48.6% had moderate sleep quality. -Routine nursing interventions were applied.	Spiritual care, sleep quality	SSCRS and PSQI.	-Participants filled out the questionnaire forms before the intervention and 4 weeks after the first session.
Senderovich et al. ; Canada; 2022.^24^	Randomizeddouble-blinded control trial	To investigate the use of TT in the management of responsive behaviors in patients with dementia.	*n* = 49 (41 females, 8 males), mean age 88.7 years -Diagnosis: severe dementia (67%), moderate (22%) and mild (10%).	Permanent residents of SageCare (a long-term care dementia institution) in Canada.	−3 phases, pretreatment, treatment, and posttreatment each lasting 5 days. -Behavior was observed and recorded by trained research assistants every 20 min during the study time.	-Control group received regular routine care. -There was also a placebo group who received mimic TT.	Frequency and intensity of the behavior symptoms of dementia, mood changes and suspected depression.	-ABRS and RMBC for behavioral symptoms of dementia. -MMSE for cognitive status.	-Mimic TT: practitioners stimulated the movements of TT, not attempting to achieve inner calmness nor interact with the energy field.
Berger et al. ; Canada; 2013.^25^	Mixed-methods study	-To increase patients/families experience of quality and satisfaction with EOLC. -To determine if therapies could enhance symptom management.	*n* = 31	The Mackenzie Richmond Hill Hospital, Toronto, Canada.	-Aromatherapy and massage, Reiki, and TTouch were provided guided by an agreed standard of practice. -VCT were taught to provide massage and received supervision from the CTC to support therapeutic relationship.	No control group.	Pain, discomfort, depression, anxiety, stiff, not relaxed (restless or tense), total improvement, inner stillness or peace.	Patients and families rate symptoms before and after one or two sessions using VAS (from 0 = as good as it can be to 10 = as bad as it can be).	No demographic or clinical data.
Thrane et al. ; United States of America; 2017.^26^	Pre–post mixed-methods single group pilot study	To examine feasibility, acceptability, and key outcomes using Reiki therapy with children receiving PC.	*n* = 32 −16 children (8 males, 8 females, mean age 12.6 years), −15 mothers (mean age 43.7 years) -1 nurse (female)	Children recruited from Supportive Care Service of Children’s Hospital, University of Pittsburgh Medical Center, Pennsylvania.	-Two 24-min Reiki sessions (done by a pediatric nurse with 12 years of experience) -Minimum of 1 and maximum of 3 days between sessions.	No control group.	Pain, anxiety and relaxation	-Pain (VAS or Wong-Baker faces Pain Scale) -Anxiety and relaxation (HR and RR).	Children diagnosis: 44% cancer, 25% congenital condition, 31% genetic condition.

ABRS: Modified Agitated Behavior Rating Scale; AFF: Analgesic Follow-up Form; BFI: Brief Fatigue Inventory; CTC: Complementary Therapy Consultant; ECOG-PS: Eastern Cooperative Oncology Group Performance Status Scale; EOLC: End-of-Life Care; FLACC: Faces-Legs-Activity-Cry-Consolability; HR: Heart Rate; MMSE: Mini-Mental State Exam; NPRS: Numeric Pain Rating Scale; NSAID: Non-Steroid Anti-Inflammatory Drug(s); PC: Palliative Care; PROMIS-29: Patient Reported Outcomes Measurement Information System; PSQI: Pittsburgh Sleep Quality Index; PTES: Perceived Treatment Efficacy Scale; QOL: Quality of life; RMBC: Revised Memory and Behavior Check; RR: Respiratory Rate; SSCRS: Spirituality and Spiritual Care Rating Scale; TTouch: Therapeutic Touch; VAS: Visual Analog Scale; VCT: Volunteer Complementary Therapist(s).

**Table 2.** Summary of reported outcomes in the included studies (*n* = 9).

Study	Reported improvements or favorable findings	Outcomes with no statistically significant differences
Kirshbaum et al. 2016^18^	Perceived reductions in stress/anxiety and pain, and improved sleep and emotional well-being	Not applicable (qualitative study)
Thrane et al. 2021^19^	Feasibility and acceptability of the intervention; parents reported calmness and relaxation in children, reduced pain and better quality of sleep.	Physiological parameters (heart rate, respiratory rate, oxygen saturation)
Thrane et al. 2021^20^	High acceptability of intervention; participants reported relaxation and perceived pain relief	Not applicable (single-arm feasibility study)
Thrane et al. 2022^21^	Stress reduction and improvement in the physical health domain of quality of life	Pain and physiological parameters (heart rate, respiratory rate, oxygen saturation)
Utli et al. 2023^22^	Reduced pain, fatigue, and analgesic use	None reported
Ünal Aslan and Çetinkaya 2022^23^	Improved spirituality scores and sleep quality	Religiosity; correlation between sleep quality and spirituality
Senderovich et al. 2022^24^	Reduced behavioral symptoms of dementia during treatment	Behavioral symptoms after treatment
Berger et al. 2013^25^	Patient-reported improvements in pain, anxiety, mood, restlessness, and inner peace	Not applicable (no control group)
Thrane et al. 2017^26^	Reduced respiratory rate in the verbal subgroup during treatment	Pain, anxiety, and heart rate

**Table 3.** Risk of bias for randomized studies (*n* = 3).

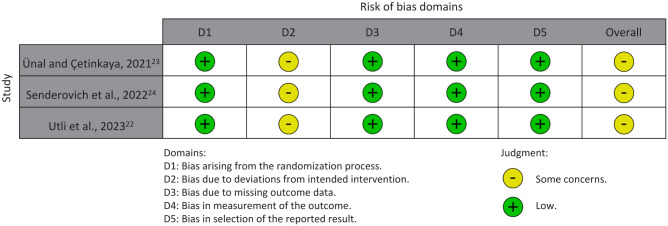

**Table 4.** Risk of bias for mixed-methods studies (*n* = 5).

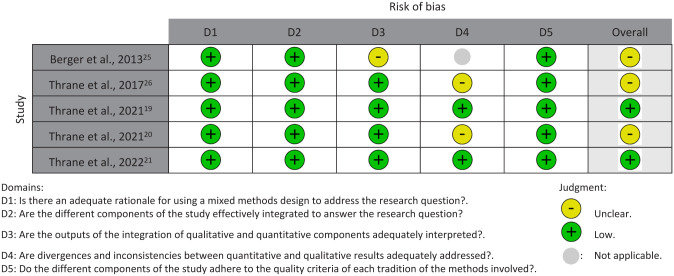

**Table 5.** Certainty of evidence for quantitative outcomes and qualitative findings.

Outcome/Review finding	Evidence type	Contributing studies	Assessment (GRADE/CERQual domains)	Certainty/confidence
Symptom burden (pain, anxiety/depression, stress, fatigue)	Quantitative (GRADE)	Kirshbaum et al.^18^, Thrane et al.^19^, Thrane et al.^20^, Thrane et al.^21^, Utli et al.^22^, Senderovich et al.^24^, Berger et al.^25^, Thrane et al.^26^	Downgraded for serious risk of bias, serious inconsistency, serious indirectness, very serious imprecision, and suspected publication bias	Very low certainty
Quality of life (sleep, emotional well-being, relaxation)		Thrane et al.^21^, Ünal Aslan and Çetinkaya^23^, Berger et al.^25^		
Perceived relaxation or calmness following sessions	Qualitative (GRADE-CERQual)	Kirshbaum et al.^18^, Thrane et al.^19^, Thrane et al.^20^, Thrane et al.^21^, Berger et al.^25^	Moderate concerns regarding methodological limitations and adequacy of data; minor concerns regarding coherence and relevance	Low confidence
Perceived emotional comfort or coping support		Kirshbaum et al.^18^, Thrane et al.^20^, Berger et al.^25^		
Caregiver-perceived patient calmness or comfort		Thrane et al.^19^, Thrane et al.^21^, Berger et al.^25^	Moderate concerns regarding methodological limitations; moderate concerns regarding coherence; serious concerns regarding adequacy of data; minor concerns regarding relevance	Very low confidence

GRADE: Grading of Recommendations Assessment, Development and Evaluation; GRADE-CERQual: Confidence in the Evidence from Reviews of Qualitative research.

### Certainty assessment

Certainty assessments were performed independently by two reviewers, with disagreements resolved through discussion.

The certainty of evidence for quantitative outcomes was assessed using the Grading of Recommendations Assessment, Development and Evaluation (GRADE) approach.^15^ Certainty was evaluated at the outcome level for symptom burden and quality of life, considering the domains of risk of bias, inconsistency, indirectness, imprecision, and publication bias. Because the included studies were heterogeneous in design, populations, interventions, and outcome measures, and no meta-analysis was performed, certainty was judged narratively across contributing studies for each outcome domain.

Confidence in qualitative findings was assessed using the Confidence in the Evidence from Reviews of Qualitative research (GRADE-CERQual) approach,^16,17^ considering methodological limitations, coherence, adequacy of data, and relevance of the contributing studies.

## Results

### Study selection

**Figure 1.**
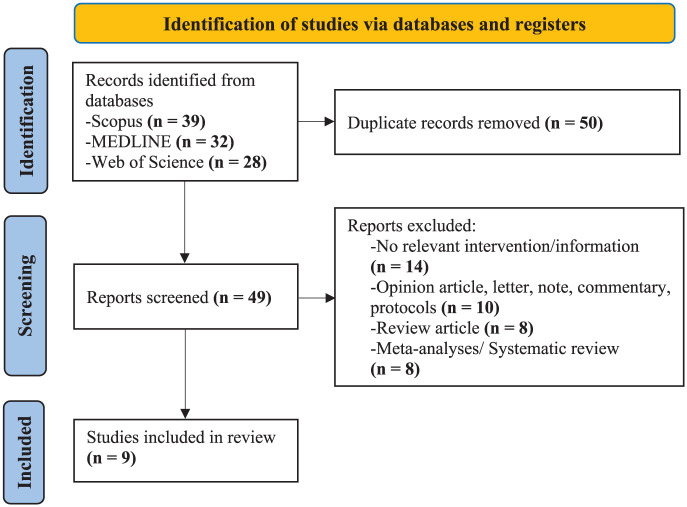
Flow diagram of the study selection process.

A total of 99 records were identified through database searches. After removal of 50 duplicates, 49 records remained for title and abstract screening. Of these, 40 records were excluded for the following reasons: studies lacking relevant interventions or information (*n* = 14); opinion articles, letters, notes, commentaries, and protocols (*n* = 10); review articles (*n* = 8); and meta-analyses or systematic reviews (*n* = 8; Supplemental Table 2). Ultimately, nine studies met the inclusion criteria for this review.^18–26^ The study selection process is shown in Figure 1.

### Study characteristics

This review included five mixed-methods studies, three randomized controlled trials, and one cross-sectional qualitative study (framework analysis).^18^ Among the mixed-methods studies, only two applied a systematic qualitative approach: one used thematic analysis^20^ and another used narrative analysis.^25^ The remaining studies incorporated qualitative data in a limited and primarily descriptive manner, without explicit use of a formal qualitative method.^19,21,26^

The studies originated from North America (United States of America, *n* = 4; Canada, *n* = 2) and Europe (Turkey, *n* = 2; United Kingdom, *n* = 1).

Across the nine studies, a total of 415 participants were reported: 220 females, 164 males, and 31 patients for whom no demographic data were provided. The overall sample comprised 351 adults and 64 children. Although the four studies by Thrane et al. were conducted in the same pediatric palliative care service, the characteristics of the child participants differ across publications, suggesting that the pediatric samples are not identical. However, potential overlap may exist among caregiver participants: two studies reported samples including 16 mothers,^20,21^ while two others reported 15 mothers of patients and one caregiver nurse.^19,26^ These publications were therefore considered as potentially reporting overlapping caregiver samples, and participants were not counted multiple times in the overall totals.

Cancer was the most prevalent diagnosis across all studies, affecting both adult and pediatric populations.^18–22^ Among the pediatric patients, some also had congenital or genetic conditions.^19,20^

The characteristics of the included studies are summarized in Table 1.

### Risk of bias in studies

Overall, the included studies presented several methodological limitations, most commonly related to lack of blinding, small sample sizes, reliance on subjective outcome measures, and absence of control groups in non-randomized designs. These factors may increase the risk of performance, detection, and reporting biases across the body of evidence.

The overall risk of bias in the randomized controlled trials was rated as “some concerns” (Table 3). In all three randomized controlled trials, the allocation sequence was generated using methods such as computer-generated random numbers or sealed envelopes, suggesting a low risk of selection bias.^22–24^

Utli et al.^22^ employed a single-blinded design in which participants were unaware of group allocation; however, the researchers were not blinded, introducing potential performance and detection bias. In addition, expectation bias cannot be excluded, as participants receiving Reiki or acupressure may have anticipated therapeutic benefits. Although analgesic intake was monitored, variability in concomitant medication use may also represent a potential confounding factor.^22^

In the trial conducted by Ünal Aslan and Çetinkaya,^23^ detection bias is possible because outcomes such as spirituality and sleep quality were assessed using self-reported scales that may be influenced by participant expectations or perceptions.

Senderovich et al.^24^ conducted the only double-blinded trial; however, some methodological concerns remain. The observed effects were limited to the period of therapy application, and no long-term follow-up was performed to assess sustained benefits. In addition, maintaining true double blinding in studies involving Therapeutic Touch may be challenging because the intervention requires direct interaction between practitioner and patient.^24^

When mixed-methods studies were appraised (Table 4), three studies raised concerns due to methodological limitations that may introduce bias.^19,25,26^

Thrane et al.^19^ evaluated Reiki delivered in a home setting, which may increase susceptibility to performance bias due to therapist presence and the informal environment. Social desirability bias may also have influenced parental reports of perceived benefits, and the absence of standardized physiological outcome measures limits the objectivity of the findings.^19^

Berger et al.^25^ relied largely on descriptive and anecdotal reporting, using subjective patient and family accounts without comparative measures. The small and potentially non-representative sample limits the generalizability of the findings, and the authors’ direct involvement in the program introduces the possibility of institutional or investigator bias.^25^

In the study by Thrane et al.,^26^ methodological concerns include the small, non-randomized sample and reliance on subjective assessments, particularly in nonverbal children. Furthermore, the principal investigator delivered the intervention, which may increase the risk of researcher bias. The absence of a control group also makes it difficult to attribute observed outcomes specifically to Reiki.^26^

Although both studies by Thrane et al.^20,21^ were rated as having relatively low risk of bias according to the appraisal tool, several methodological limitations remain. These include potential selection bias, lack of a control group, reliance on subjective outcome measures, and investigator involvement in intervention delivery, which complicate attempts to isolate the specific effects of Reiki.^20,21^

Finally, the qualitative study by Kirshbaum et al.^18^ was judged to be of high methodological quality, meeting eight of the ten appraisal criteria. Nevertheless, potential sources of bias should be considered. Participants were self-selected and may have been more receptive to complementary therapies, introducing possible selection bias. In addition, retrospective interviews may be subject to recall and response bias, and the absence of a comparison group limits the ability to attribute reported experiences specifically to Reiki.^18^

Taken together, these methodological limitations should be considered when interpreting the reported benefits of Reiki and Therapeutic Touch in palliative care settings.

### Results of individual studies

The main findings of the included studies are summarized in Table 2.

Outcomes are presented as reported in the original studies. Given the methodological limitations, heterogeneity of designs, and small sample sizes across the included studies, these findings should be interpreted with caution.

### Synthesis of results

#### Quantitative outcomes

##### Symptom management/burden

Eight of the nine included studies assessed symptom-related outcomes. The most frequently evaluated domains were pain, anxiety or depression, stress, fatigue, and overall symptom burden.

Across studies, some reported improvements in pain and related symptoms following Reiki or Therapeutic Touch interventions. These findings were described in adult patients with advanced cancer as well as in pediatric populations receiving palliative care.^18–22^

Reductions in anxiety, depression, or emotional distress were also reported in two studies.^18,24^ However, results were not consistent across all studies. In particular, some investigations reported trends toward improvement without reaching statistical significance.^21,26^ In addition, several studies relied on subjective outcome measures or lacked control groups, which limits the strength of the conclusions that can be drawn.

Improvements in related domains such as stress,^21^ fatigue,^22^ or overall symptom burden^25^ were also reported in some studies.

##### Quality of life

Quality of life outcomes were assessed in six of the nine included studies using various instruments and domains, including sleep quality, relaxation, emotional well-being, energy, hope, and self-confidence.

Some studies reported improvements in aspects of quality of life following Reiki or Therapeutic Touch interventions, particularly in domains such as sleep quality, relaxation, and emotional well-being.^21,23,25^ However, these findings were not consistent across all studies. In addition, the heterogeneity of outcome measures and the methodological limitations of several studies—including small sample sizes and the absence of control groups in some designs—limit the interpretation of these results.

The remaining studies assessing quality of life did not observe clear or statistically significant differences between the intervention and comparison groups.^18,20,24^

#### Qualitative outcomes

Five studies reported qualitative findings related to participants’ experiences of the interventions.

Across these studies, participants frequently described the interventions as calming and relaxing, with perceived improvements in comfort, emotional well-being, and coping with illness.^18,20,25^ Family members and caregivers also reported observing increased calmness and relaxation among patients following the sessions.^19,21^

These qualitative observations provide insight into patient and caregiver experiences but should be interpreted cautiously given the small samples, exploratory study designs, and lack of control conditions in most studies.

#### Adverse events

Reporting of adverse events was limited across the included studies.

Only one study explicitly monitored adverse events.^19^ Thrane et al. reported one serious adverse event rated as potentially study-related: the child became agitated and developed tachypnea, bradycardia, and low oxygen saturation; the episode resolved with lavage and suctioning, and the child was later treated with antibiotics for a respiratory infection. The same study also reported two unrelated adverse events: one child died from the underlying illness, and another had eye deviation due to nerve palsy.^19^

Most of the remaining studies did not systematically assess or report harms, although some described the interventions as acceptable or well tolerated. Consequently, the absence of reported harms should not be interpreted as evidence of safety but rather reflects the limited reporting of adverse events in the available literature.

### Certainty of evidence

Overall, the certainty of evidence for both symptom burden and quality-of-life outcomes was rated as very low (Table 5). The certainty assessment for quality-of-life outcomes was based on the three studies that reported quantitative measures suitable for evaluation.

Evidence was downgraded due to several limitations. Risk of bias was identified in multiple studies because of lack of blinding, small sample sizes, and uncontrolled designs. Inconsistency reflects heterogeneity in interventions and outcome measures across studies. Indirectness arises from variation in populations and outcome domains, while imprecision reflects small samples and exploratory study designs. Publication bias cannot be excluded given the limited number of heterogeneous studies.

Confidence in the qualitative findings ranged from low to very low (Table 5). These ratings reflect methodological limitations, including small samples and exploratory study designs, as well as concerns regarding the adequacy of qualitative data in several mixed-methods studies.

Taken together, these assessments indicate that the currently available evidence remains limited and should be interpreted cautiously.

## Discussion

### Summary of findings

This systematic review examined the available evidence on Reiki and Therapeutic Touch for symptom burden and quality of life in palliative and end-of-life care. Across the nine included studies (415 participants), cancer was the predominant diagnosis, although some pediatric populations included congenital or genetic conditions.

Some studies reported improvements in symptoms such as pain, anxiety, depression, fatigue, and stress following Reiki or Therapeutic Touch interventions. Improvements in domains related to quality of life—including sleep, relaxation, emotional well-being, energy, and hope—were also described in several studies, while qualitative findings highlighted participants’ perceptions of relaxation, comfort, and emotional support. However, the certainty of evidence for both symptom burden and quality-of-life outcomes was rated as very low, and confidence in qualitative findings ranged from low to very low. These ratings reflect important methodological limitations, including small sample sizes, heterogeneous outcome measures, lack of control groups in several studies, and reliance on subjective outcomes.

Potential harms or adverse events were rarely assessed or reported in the included studies. Therefore, the absence of reported harms should not be interpreted as evidence of safety but rather reflects limited monitoring and reporting in the current literature.

Overall, while some studies reported perceived improvements in symptoms and well-being, the limited number of studies and methodological weaknesses prevent firm conclusions regarding the effectiveness of Reiki or Therapeutic Touch in palliative care.

### General interpretation of the results

Before interpreting the findings, it is important to note that in all included studies Reiki and Therapeutic Touch were delivered as complementary interventions alongside standard pharmacological treatment rather than as substitutes for conventional care. This context is relevant because concurrent medical treatments may have influenced the observed outcomes. In addition, the absence of blinding—an inherent challenge for interventions such as Reiki and Therapeutic Touch—and the reliance on subjective outcome measures increase the potential for expectation and reporting bias. Within patient-centered models of care, complementary therapies are sometimes incorporated to address multidimensional needs in palliative care, including physical, psychological, and spiritual domains.^8^

Across the included studies, some improvements were reported in symptom domains such as pain, anxiety, fatigue, and perceived well-being. Similar observations have been reported in earlier reviews of biofield therapies, including that of Henneghan and Schnyer,^11^ although previous syntheses frequently combined multiple modalities and lacked formal methodological appraisal. Other reviews and analyses have also described improvements in symptoms such as pain, anxiety, depression, fatigue, mood disturbance, or nausea following Reiki or related biofield therapies,^1,3,27–29^ while Cochrane reviews of Therapeutic Touch have suggested possible reductions in pain intensity despite limited and heterogeneous evidence.^30^

Quality-of-life findings were more variable, reflecting both methodological differences and the multidimensional nature of quality of life in palliative care. Domains such as physical comfort, emotional well-being, independence, social relationships, and spiritual well-being have been identified as central components of quality of life in palliative settings.^31^ In several studies included in this review, improvements were reported in domains such as sleep quality, relaxation, emotional well-being, or analgesic use,^22,23,25^ although these results should be interpreted cautiously given the reliance on subjective measures and the potential influence of contextual or placebo effects. Related research has also explored potential mechanisms involving relaxation responses, autonomic regulation, and emotional processing in Reiki practice,^32,33^ and observational or exploratory studies have reported improvements in affect, symptoms, or well-being following Reiki sessions.^34–36^ In specific clinical contexts, complementary therapies such as Therapeutic Touch have also been associated with improvements in agitation, spiritual well-being, or sleep quality in certain patient groups.^23,24,37^

Taken together, the available literature suggests that Reiki and Therapeutic Touch may be associated with perceived improvements in some symptom and well-being domains in palliative care settings. However, the small number of studies, methodological variability, and very low certainty of evidence preclude firm conclusions regarding their effectiveness.

At present, the available evidence should therefore be interpreted cautiously. Further well-designed trials with larger samples and standardized outcome measures are needed to clarify the potential role of these interventions within palliative care.

### Strengths and limitations

This systematic review has several strengths. It focuses specifically on Reiki and Therapeutic Touch in palliative and end-of-life care, an area that remains relatively underexplored. The inclusion of randomized controlled trials, mixed-methods studies, and qualitative research allowed both quantitative outcomes and patient-reported experiences to be considered. In addition, a structured critical appraisal using established methodological tools and the application of GRADE and GRADE-CERQual frameworks provided a transparent assessment of the certainty of the evidence. The search strategy covered three major databases over 11 years, and both physical and psychosocial outcomes were examined, reflecting the multidimensional nature of palliative care.

However, several important limitations must be acknowledged. The evidence base itself is limited and heterogeneous, comprising a small number of studies with varied designs, populations, and outcome measures. Many studies included small samples, short follow-up periods, and subjective outcome measures, and several lacked control groups or adequate blinding procedures, increasing the risk of bias. In addition, the subjective nature of many outcomes raises the possibility that some reported improvements may reflect contextual or placebo effects related to therapeutic interaction, relaxation, or participants’ expectations. Accordingly, the certainty of evidence for quantitative outcomes was rated as very low, and confidence in qualitative findings ranged from low to very low.

Additional methodological limitations relate to the review process. The search strategy was limited to three databases and English-language publications, and grey literature was not included, which may have reduced comprehensiveness and introduced publication or language bias. The review also focused on studies published between 2013 and 2024 in order to update a previous review covering earlier years,^11^ meaning that earlier literature was not reassessed within the present analysis.

Potential harms or adverse events were rarely assessed or reported in the included studies. Consequently, the absence of reported harms should not be interpreted as evidence of safety but rather reflects the limited monitoring and reporting of adverse events in the available literature.

Finally, PROSPERO registration occurred after the search and screening stages, although it preceded data extraction and analysis. In addition, adverse events or harms were not specified as predefined outcomes in the protocol; however, during manuscript revision the included studies were re-examined for any reporting of adverse events or safety outcomes associated with the interventions. Similarly, formal certainty-of-evidence assessments using the GRADE approach for quantitative findings and the GRADE-CERQual framework for qualitative findings were introduced during manuscript revision to provide a structured evaluation of the certainty of the available evidence.

Taken together, these limitations highlight that the current evidence base for Reiki and Therapeutic Touch in palliative care remains limited and should be interpreted cautiously.

### Implications for practice, policy, and future research

From a clinical perspective, Reiki and Therapeutic Touch may be considered within broader holistic or integrative care approaches when offered as complementary interventions alongside standard medical treatment. Their reported acceptability and feasibility in several studies suggest that they may be of interest to some patients and caregivers seeking supportive care options. However, their use should be guided by careful clinical judgment and transparent communication regarding the limited evidence base.

At the policy level, the current evidence does not support definitive recommendations regarding the routine integration of these therapies into palliative care services. Instead, the findings highlight the need for further research to better understand their potential role within palliative care.

Future research should aim to address the substantial limitations of the existing literature, including small sample sizes, heterogeneous study designs, and reliance on subjective outcome measures. Adequately powered randomized controlled trials with standardized interventions and validated outcome measures are needed. Future studies should also ensure systematic reporting of adverse events and harms, as these were rarely assessed in the current literature. In addition, qualitative research may help further explore patients’ and caregivers’ experiences and expectations regarding complementary therapies in palliative care.

## Conclusions

This systematic review examined the available evidence regarding Reiki and Therapeutic Touch in palliative and end-of-life care. Some studies reported improvements in symptoms such as pain, anxiety, depression, fatigue, stress, and perceived well-being, as well as in domains related to quality of life including sleep, relaxation, hope, and emotional comfort.

However, the overall evidence base remains limited and heterogeneous. The included studies were generally small, used diverse outcome measures, and often relied on subjective assessments, resulting in very low certainty of evidence. Consequently, the current evidence does not allow firm conclusions regarding the effectiveness of Reiki or Therapeutic Touch in palliative care.

Potential harms or adverse events were rarely assessed or reported in the included studies. Therefore, the absence of reported harms should not be interpreted as evidence of safety but rather reflects the limited monitoring and reporting of adverse events in the available literature.

Further rigorous research, including adequately powered studies with standardized outcome measures and systematic reporting of both benefits and harms, is needed to clarify the potential role of these interventions in palliative care.

## Supplemental Material

sj-docx-1-pmj-10.1177_02692163261437606 – Supplemental material for Reiki and Therapeutic Touch for symptom burden and quality of life in palliative settings: A systematic review Supplemental material, sj-docx-1-pmj-10.1177_02692163261437606 for Reiki and Therapeutic Touch for symptom burden and quality of life in palliative settings: A systematic review by Raquel Pontes-Gomes and Paulo Reis-Pina in Palliative Medicine

sj-docx-2-pmj-10.1177_02692163261437606 – Supplemental material for Reiki and Therapeutic Touch for symptom burden and quality of life in palliative settings: A systematic review Supplemental material, sj-docx-2-pmj-10.1177_02692163261437606 for Reiki and Therapeutic Touch for symptom burden and quality of life in palliative settings: A systematic review by Raquel Pontes-Gomes and Paulo Reis-Pina in Palliative Medicine
