# Supplementary material for: Reiki and Therapeutic Touch for symptom burden and quality of life in palliative settings: A systematic review
Source: Palliat Med. 2026 May 10;40(7):958–70. doi: 10.1177/02692163261437606 (PMC13365623; doi:10.1177/02692163261437606)
Supplement: sj-docx-1-pmj-10.1177_02692163261437606 – Supplemental material for Reiki and Therapeutic Touch for symptom burden and quality of life in palliative settings: A systematic review [file sj-docx-1-pmj-10.1177_02692163261437606.docx]

**Supplementary Table 1 - Literature search strategy**

| **Database** | **Search strategy** |
| --- | --- |
| MEDLINE (via Pubmed) | - ("palliative care"[All Fields] OR "terminal care"[All Fields] OR "hospice care"[All Fields] OR "end of life care"[All Fields]) AND - ("therap* touch"[All Fields] OR ("therapeutic touch"[MeSH Terms] OR ("therapeutic"[All Fields] AND "touch"[All Fields]) OR "therapeutic touch"[All Fields] OR "reiki"[All Fields])) AND - Filters: English, MEDLINE, from 2013/1/1 - 2024/12/31 |
| Scopus | - TITLE-ABS-KEY ( ( "palliative care" OR "terminal care" OR "hospice care" OR "end of life care" ) ) AND - TITLE-ABS-KEY ( ( "therap* touch" OR "reiki" ) ) AND - LANGUAGE ( English ) AND - PUBYEAR > 2012 AND PUBYEAR < 2025 |
| Web of Science (Core Collection) | - "palliative care" OR "terminal care" OR "hospice care" OR "end of life care" (Topic) AND - "therap* touch” OR "reiki" (Topic) AND - English (Language) AND - 2013-01-01/2024-12-31 (Publication Date) |
