# Supplementary material for: Reiki and Therapeutic Touch for symptom burden and quality of life in palliative settings: A systematic review
Source: Palliat Med. 2026 May 10;40(7):958–70. doi: 10.1177/02692163261437606 (PMC13365623; doi:10.1177/02692163261437606)
Supplement: sj-docx-2-pmj-10.1177_02692163261437606 – Supplemental material for Reiki and Therapeutic Touch for symptom burden and quality of life in palliative settings: A systematic review [file sj-docx-2-pmj-10.1177_02692163261437606.docx]

**Supplemental Table 2 - Rationale for exclusion of full-text articles (n=40)**

| **Authors; year** | **Study design** | **Reason for exclusion** |
| --- | --- | --- |
| Dingley et al; 2021 [6] | Integrative review | Study design |
| Lopes-Júnior et al; 2021 [7] | Systematic review | Study design |
| Henneghan and Schnyer; 2015 [11] | Narrative review | Study design |
| Marcus; 2013 [38] | Review | Study design |
| Gonella et al; 2014 [39] | Review | Study design |
| Rera et al; 2018 [40] | Review | Study design |
| Booth et al; 2018 [41] | Scoping review | Study design |
| Billot et al; 2019 [42] | Review | Study design |
| Frenkel et al; 2020 [43] | Narrative review | Study design |
| Hammerschlag et al; 2014 [44] | Systematic review | Study design |
| Diop et al; 2017 [45] | Systematic review and Meta-Analysis | Study design |
| Zeng et al; 2018 [46] | Systematic review | Study design |
| Shepperd et al; 2021 [47] | Systematic review | Study design |
| Quigley and McCleskey; 2021 [48] | Systematic review | Study design |
| Mitchinson et al; 2022 [49] | Systematic review | Study design |
| Guo et al; 2024 [50] | Meta-analyses | Study design |
| Demir et al; 2013 [51] | Protocol | Study design |
| Komatz and Carter; 2015 [52] | Commentary | Study design |
| Rousseau; 2016 [53] | Commentary/personal narrative | Study design |
| Krajnik; 2017 [54] | Short note | Study design |
| Weaver; 2017 [55] | Perspective article/ opinion | Study design |
| Milán; 2020 [56] | Essay/personal reflection | Study design |
| Karaderi et al; 2020 [57] | Perspective article/ opinion | Study design |
| Kaye et al; 2020 [58] | Note | Study design |
| Lion; 2020 [59] | Opinion | Study design |
| Baptista Peixoto Befecadu et al; 2023 [60] | Multicenter longitudinal mixed-methods protocol study | Study design |
| Kaneria; 2014 [61] | Case report | No relevant intervention |
| Houck; 2014 [62] | Educational intervention | No relevant intervention |
| Wolf et al; 2015 [63] | Commentary | No relevant information |
| Senderovich et al; 2016 [64] | Retrospective review | Retrospective chart review based on client characteristics and practitioners' observations. |
| Litzelman et al; 2016 [65] | Cross-sectional study | No relevant intervention |
| López Pardo et al; 2017 [66] | Case report | No relevant information |
| McNiel and Westphal; 2018 [67] | Qualitative study | No relevant intervention |
| Plymire et al; 2018 [68] | Retrospective review | Retrospective Review of Limitations of Care |
| McGahan et al; 2020 [69] | Descriptive report | No relevant information |
| Gaille et al; 2020 [70] | Ethical and social reflection | No relevant information |
| Parent et al; 2020 [71] | Conceptual reflection | No relevant information |
| Wang et al; 2023 [72] | Retrospective observational study | No relevant information |
| Decavèle et al; 2023 [73] | Clinical trial | No relevant information |
| Browning et al; 2024 [74] | Intervention project | Educational project |

**References:**

6. Dingley C, Ruckdeschel A, Kotula K, Lekhak N. Implementation and outcomes of complementary therapies in hospice care: an integrative review. Palliat Care Soc Pract. 2021;15:26323524211051753. doi:10.1177/26323524211051753.

7. Lopes-Júnior LC, Urbano IR, Schuab SIPC, Pessanha RM, Rosa GS, Lima RAG. Effectiveness of complementary therapies for the management of symptom clusters in palliative care in pediatric oncology: a systematic review. Rev Esc Enferm USP. 2021;55:e03709. doi:10.1590/S1980-220X2020025103709.

11. Henneghan AM, Schnyer RN. Biofield therapies for symptom management in palliative and end-of-life care. Am J Hosp Palliat Care. 2015;32(1):90–100. doi:10.1177/1049909113509400.

38. Marcus DA. The role of volunteer services at cancer centers. Curr Pain Headache Rep. 2013;17(11):376. doi:10.1007/s11916-013-0376-1.

39. Gonella S, Garrino L, Dimonte V. Biofield therapies and cancer-related symptoms: a review. Clin J Oncol Nurs. 2014;18:568–76. doi:10.1188/14.CJON.568-576.

40. Rera M, Vallot C, Lefrançois C. The Smurf transition: new insights on ageing from end-of-life studies in animal models. Curr Opin Oncol. 2018;30:38–44. doi:10.1097/CCO.0000000000000419.

41. Booth A, Maddison J, Wright K, Fraser L, Beresford B. Research prioritisation exercises related to the care of children and young people with life-limiting conditions, their parents and all those who care for them: a systematic scoping review. Palliat Med. 2018;32:1552–66. doi:10.1177/0269216318800172.

42. Billot M, Daycard M, Wood C, Tchalla A. Reiki therapy for pain, anxiety and quality of life. BMJ Support Palliat Care. 2019;9:434–8. doi:10.1136/bmjspcare-2019-001775.

43. Frenkel M, Sapire K, Lacey J, Sierpina VS. Integrative medicine: adjunctive element or essential ingredient in palliative and supportive cancer care? J Altern Complement Med. 2020;26:779–83. doi:10.1089/acm.2019.0316.

44. Hammerschlag R, Marx BL, Aickin M. Nontouch biofield therapy: a systematic review of human randomized controlled trials reporting use of only nonphysical contact treatment. J Altern Complement Med. 2014;20:881–92. doi:10.1089/acm.2014.0017.

45. Diop MS, Rudolph JL, Zimmerman KM, Richter MA, Skarf LM. Palliative care interventions for patients with heart failure: a systematic review and meta-analysis. J Palliat Med. 2017;20:84–92. doi:10.1089/jpm.2016.0330.

46. Zeng YS, Wang C, Ward KE, Hume AL. Complementary and alternative medicine in hospice and palliative care: a systematic review. J Pain Symptom Manage. 2018;56:781–94.e4. doi:10.1016/j.jpainsymman.2018.07.016.

47. Shepperd S, Gonçalves-Bradley DC, Straus SE, Wee B. Hospital at home: home-based end-of-life care. Cochrane Database Syst Rev. 2021;3:CD009231. doi:10.1002/14651858.CD009231.pub3.

48. Quigley DD, McCleskey SG. Improving care experiences for patients and caregivers at end of life: a systematic review. Am J Hosp Palliat Care. 2021;38:84–93. doi:10.1177/1049909120931468.

49. Mitchinson L, Chu C, Bruun A, Sisk AR, Armstrong M, Vindrola-Padros C, et al. How best to capture the impact of complementary therapies in palliative care: a systematic review to identify and assess the appropriateness and validity of multi-domain tools. Palliat Med. 2022;36:1320–35. doi:10.1177/02692163221122955.

50. Guo X, Long Y, Qin Z, Fan Y. Therapeutic effects of reiki on interventions for anxiety: a meta-analysis. BMC Palliat Care. 2024;23:147. doi:10.1186/s12904-024-01439-x.

51. Demir M, Can G, Celek E. Effect of reiki on symptom management in oncology. Asian Pac J Cancer Prev. 2013;14:4931–3. doi:10.7314/apjcp.2013.14.8.4931.

52. Komatz K, Carter B. Pain and symptom management in pediatric palliative care. Pediatr Rev. 2015;36:527–34. doi:10.1542/pir.36-12-527.

53. Rousseau P. The physical exam and touch. J Am Geriatr Soc. 2016;64:645–6. doi:10.1111/jgs.13998.

54. Krajnik M. Learning to touch the patient's soul: a difficult lesson from the Netherlands. Pol Arch Intern Med. 2017;127:289–90. doi:10.20452/pamw.4014.

55. Weaver M. Healing touch: positively sharing energy in a pediatric hospital. J Pain Symptom Manage. 2017;54:259–61. doi:10.1016/j.jpainsymman.2016.12.340.

56. Milán BM. We missed losing him. Palliat Support Care. 2020;18:623–4. doi:10.1017/S1478951520000565.

57. Karaderi T, Bareke H, Kunter I, Seytanoglu A, Cagnan I, Balci D, et al. Host genetics at the intersection of autoimmunity and COVID-19: a potential key for heterogeneous COVID-19 severity. Front Immunol. 2020;11:586111. doi:10.3389/fimmu.2020.586111.

58. Kaye EC, Kegel A, Weber M, Cartwright C, Spraker-Perlman H, Ribinson GW, et al. Food is love: partnering with families to provide nourishment at the end of life. J Clin Oncol. 2020;38:1864–7. doi:10.1200/JCO.20.00176.

59. Lion AH. First rites: a spiritual history case study. JAMA Oncol. 2020;6:475–6. doi:10.1001/jamaoncol.2019.5980.

60. Baptista Peixoto Befecadu F, Stirnemann J, Guerreiro I, Fusi-Schmidhauser T, Jaksic C, Larkin PJ, et al. PANDORA dyadic project: hope, spiritual well-being and quality of life of dyads of patients with chronic obstructive pulmonary disease in Switzerland—a multicentre longitudinal mixed-methods protocol study. BMJ Open. 2023;13:e068340. doi:10.1136/bmjopen-2022-068340.

61. Kaneria A. Opioid-induced hyperalgesia: when pain killers make pain worse. BMJ Case Rep. 2014;2014:bcr2014204551. doi:10.1136/bcr-2014-204551.

62. Houck D. Helping nurses cope with grief and compassion fatigue. Clin J Oncol Nurs. 2014;18:454–8. doi:10.1188/14.CJON.454-458.

63. Wolf SM, Berlinger N, Jennings B. Forty years of work on end-of-life care: from patients' rights to systemic reform. N Engl J Med. 2015;372:678–82. doi:10.1056/NEJMms1410321.

64. Senderovich H, Ip ML, Berall A, Karuza J, Gordan M, Binns M, et al. Therapeutic touch® in a geriatric palliative care unit: a retrospective review. Complement Ther Clin Pract. 2016;24:134–8. doi:10.1016/j.ctcp.2016.06.002.

65. Litzelman K, Kent EE, Rowland JH. Social factors in informal cancer caregivers: the interrelationships among social stressors, relationship quality, and family functioning in the CanCORS data set. Cancer. 2016;122:278–86. doi:10.1002/cncr.29741.

66. Lopez Pardo P, Jiménez Rojas C, Moral Carretón M. Neuromyelitis optica and tactile and visual hallucinations in an elderly patient. Age Ageing. 2017;46:156–7. doi:10.1093/ageing/afw170.

67. McNiel P, Westphal J. Namaste care™: a person-centered care approach for Alzheimer's and advanced dementia. West J Nurs Res. 2018;40:37–51. doi:10.1177/0193945916679631.

68. Plymire CJ, Miller EG, Frizzola M. Retrospective review of limitations of care for inpatients at a free-standing, tertiary care children's hospital. Children. 2018;5:164. doi:10.3390/children5120164.

69. McGahan RK, Gafford EF, Whitson BA, Papadimos TJ, Tripathi RS. Palliative use of nitroglycerin to improve microvascular circulation. J Palliat Care. 2020;35:75–7. doi:10.1177/0825859719856563.

70. Gaille M, Araneda M, Dubost C, Guillermain C, Kaakai S, Ricadat E, et al. Ethical and social implications of approaching death prediction in humans: when the biology of ageing meets existential issues. BMC Med Ethics. 2020;21:64. doi:10.1186/s12910-020-00502-5.

71. Parent B, Gelb B, Latham S, Lewis A, Kimberly LL, Caplan AL. The ethics of testing and research of manufactured organs on brain-dead/recently deceased subjects. J Med Ethics. 2020;46:199–204. doi:10.1136/medethics-2019-105674.

72. Wang J, Foxman B, Rao K, Cassone M, Gibson K, Mody L, et al. Association of patient clinical and gut microbiota features with vancomycin-resistant enterococci environmental contamination in nursing homes: a retrospective observational study. Lancet Healthy Longev. 2023;4:e600–7. doi:10.1016/S2666-7568(23)00188-5.

73. Decavèle M, Bureau C, Campion S, Nierat MC, Rivals I, Wattiez N, et al. Interventions relieving dyspnea in intubated patients show responsiveness of the mechanical ventilation-respiratory distress observation scale. Am J Respir Crit Care Med. 2023;208:39–48. doi:10.1164/rccm.202301-0188OC.

74. Browning JS, Rosselet RM, Von Ah D, Overcash J. Educating nurses and providers on therapeutic touch® in patients with cancer to increase use in an ambulatory palliative care clinic. Clin J Oncol Nurs. 2024;28:567–74. doi:10.1188/24.CJON.567-574.
